# Blockade of 5-Hydroxytryptamine 2A Receptor Attenuates Precipitation of Naloxone-Induced Withdrawal Symptoms in Opioid-Exposed Mice

**DOI:** 10.3389/fnbeh.2021.797217

**Published:** 2022-02-09

**Authors:** Bing Li, Junyu Jiang, Li Zhou, Xinrong Tao, Qixian Sun, Jiaxin Liu, Yang Liu, Gang Pang

**Affiliations:** ^1^Center for Medical Research, School of Medicine, Anhui University of Science and Technology, Huainan, China; ^2^College of Basic Medical Sciences, Anhui Medical University, Hefei, China

**Keywords:** addiction, serotonin, 5-HT2A R, MDL100907, heroin, withdrawal, hippocampus

## Abstract

Heroin dependency has become a global problem and has caused significant clinical and socioeconomic burdens along with devastating medical consequences. Chronic drug exposure alters the expression and functional activity of 5-hydroxytryptamine (serotonin) 2A receptors (5-HT2ARs) in the brain. Furthermore, pharmacological blockade of 5-HT2ARs reduces cue-induced cocaine craving behaviors. In this study, we explored the influence of 5-HT2ARs on heroin-withdrawal behaviors in mice. Black C57BL/6J mice were given gradually increasing (10–50 mg/kg over 4.5 days) doses of heroin to induce heroin dependency, after which naloxone was given to precipitate withdrawal symptoms. MDL100907, a selective and potent 5-HT2AR antagonist, attenuated naloxone-precipitated withdrawal symptoms in these mice. In addition, 5-HT2AR protein levels increased significantly in the medial prefrontal cortex (mPFC), while phosphorylation of extracellular signal-regulated kinase (p-ERK) decreased in the mPFC after heroin exposure. In conclusion, these results suggest that 5-HT2ARs might be involved in the development of opioid dependency and that pharmacological blocking of 5-HT2ARs might be a new therapeutic strategy for heroin dependency.

## Introduction

Diacetylmorphine (heroin) is one of the most addictive drugs. Heroin use disorders have become a global problem and have caused significant clinical and socioeconomic burdens along with devastating medical consequences. As a morphine derivative, heroin has a high-addictive potency. Once it enters the brain, heroin is rapidly hydrolyzed to 6-acetylmorphine and morphine by serum cholinesterase, which then binds with opioid receptors to activate dopaminergic neurons (Inturrisi et al., 1983; Johnson and North, 1992). Heroin derivatives have a high affinity for opioid receptors in the brain. The opioid receptor superfamily consists of δ (delta), κ (kappa), and μ (mu) opioid receptors (DOR, KOR, and MOR, respectively) (Granier et al., 2012). MOR is hyperactivated after drug binding and modulates neurotransmitter efflux to reinforce addictive behaviors (Kreek et al., 2012).

Heroin addiction is increasing, especially in adolescents and young adults (Kuehn, 2013; Dart et al., 2015; Griesler et al., 2019; Kelley-Quon et al., 2019). Rehabilitation following heroin addiction takes a long time, and antidrug treatment can cause serious side effects in patients. Furthermore, deaths related to heroin overdose have been increasing at an alarming rate in the United States and Canada (Jalal et al., 2018; Banerjee et al., 2019; Dong et al., 2019; Jones et al., 2020). Heroin use can also cause long-term health complications, such as viral infections (Reardon, 2019), chronic obstructive pulmonary disease (Grischott et al., 2019; Tashkin, 2019; Nightingale et al., 2020), and cerebellar dysfunctions (Moreno-Rius, 2019).

Heroin disrupts the physiological neurotransmitter signaling cascades, especially those involving 5-hydroxytryptamine (5-HT) neurotransmitters. The 5-HT receptors modulate drug-addictive behaviors. For example, activation of 5-HT 2C receptors (5-HT2CRs) inhibits behavioral sensitization and drug dependency in heroin-treated mice (Wu et al., 2015; Zhang et al., 2016). 5-Hydroxytryptamine (serotonin) 2A receptors (5-HT2ARs) also participate in opiate addiction in rodents and humans. Recently, Odagaki et al. (2021a,b) reported that long-term exposure to heroin leads to adaptive changes in 5-HT2ARs in the human brain. 5-HT2ARs activate phospholipase C through Gα_q/11_, which contributes to inositol 1,4,5-triphosphate and 1,2-diacylglycerol accumulation, intracellular Ca^2+^ release, and protein kinase C activation (Odagaki et al., 2021a,b).

5-Hydroxytryptamine (serotonin) 2A receptors regulate neuropsychological functions (Pogorelov et al., 2017; Mora et al., 2018; Odabas-Geldiay et al., 2019) and have been shown to play a role in a number of neurological disorders, including Alzheimer’s disease (Ballard et al., 2018), Parkinson’s disease (Cummings et al., 2014; Ohno et al., 2015), obsessive–compulsive disorder (El Mansari and Blier, 2006; Steeves and Fox, 2008; Simpson et al., 2011; Sinopoli et al., 2017), schizophrenia (Girgis et al., 2020), autism spectrum disorder (Chen et al., 2013), depression (Underwood et al., 2018), anxiety (Weisstaub et al., 2006), insomnia (Teegarden et al., 2008), and obesity (Morris, 2017). The 5-HT2AR antagonist volinanserin or MDL100907 (Aventis Pharmaceuticals) has been used to treat neuropsychological dysfunctions in drug-addicted patients (Cunningham et al., 2013), and a synergism between the 5-HT2AR and 5-HT2CR affects the severity of addictive behaviors (Cunningham et al., 2013; Perez-Aguilar et al., 2014; Prabhakaran et al., 2015; Felsing et al., 2018). 5-HT2ARs are ubiquitously expressed in all brain regions, with the highest receptor densities in the frontal and motor cortices (Price et al., 2019). The dorsomedial prefrontal cortex (PFC), specifically the prelimbic cortical region, plays a pivotal role in drug addiction reinforcement behaviors in rodents (Gourley and Taylor, 2016). Overexpression of 5-HT2ARs has been associated with chronic dependency on opioids, worsening withdrawal symptoms, and relapse behavior after heroin exposure. These effects may have been mediated by downregulation of ERK/mitogen-activated protein kinase signaling in the PFC (Pang et al., 2016).

In this study, we aimed to investigate the impacts of 5-HT2AR antagonists on heroin-withdrawal symptoms in mice. We showed that the selective 5-HT2AR antagonist, MDL100907 (volinanserin), significantly inhibits heroin-induced abnormal motor activities and withdrawal behavior in male mice.

## Materials and Methods

### Animals

Male C57BL/6J adult mice, aged 8–12 weeks old [license # SCXY (Su) 2011-0003] and weighing 20 ± 2 g, were purchased from Cavion (Cavion Experimental Animal Co., Changzhou, China). Mice were housed in groups of four in 29 cm × 18 cm × 12 cm polycarbonate cages with *ad libitum* access to water and food under a controlled temperature (23 ± 1°C) and 12-h light/dark cycle (dark phase 7:00 p.m. to 7:00 a.m.). Only male mice were used in this study to avoid the effects of the female estrous cycle on behavioral parameters. All animal procedures followed the National Institutes of Health guidelines for the care and use of research rodents, and the Institutional Animal Care and Use Committee reviewed and approved the study protocol.

### Chemicals and Reagents

Heroin was provided by the Anhui provincial public security department (Hefei, China). MDL100907 (Griesler et al., 2019) was purchased from Sigma-Aldrich (Sigma-Aldrich, United States) and was dissolved in dimethyl sulfoxide (DMSO) to prepare different doses (i.e., 0.01, 0.03, 0.1, and 0.3 mg/kg) for intraperitoneal (IP) injection. The final concentrations of DMSO of these four doses were 0.6, 0.2, 0.06, and 0.02%, respectively.

Naloxone and saline were purchased from The First Affiliated Hospital of Anhui University of Science and Technology (Huainan, China). The time points of MDL100907 treatment were determined based on a previously published report (Wu et al., 2015), and doses were optimized in our laboratory. Naloxone (5 mg/kg) was administered by IP injection. To minimize background interference of heroin on naloxone binding to various opioid receptors and spontaneous withdrawal precipitation, naloxone was injected 2 h after heroin was administered (Wu et al., 2015). Naloxone was dissolved in 0.9% saline solution and administered at a dose of 5 mg/kg. Saline solution was used as a placebo.

For immunoblotting, we used anti-5-HT2AR (ab16028, Abcam, United States) and anti-phospho-ERK1/2 (Cell Signaling Technology, United States) primary antibodies and a horseradish peroxidase (HRP)-conjugated anti-rabbit IgG secondary antibody (SA00001-2, Proteintech, United States). Protein was quantified using a bicinchoninic acid (BCA) assay kit provided by the Beyotime Institute of Biotechnology (Haimen, China).

### Basal Locomotor Activity Recording With an Open-Field Test

The open-field test was conducted as described elsewhere (Pang et al., 2016). Briefly, mice were placed in the center of a white open field with dimensions of 30 cm × 30 cm × 37.5 cm. The movement and activities of the mice were tracked by a video camera equipped with EthoVision-XT-5.1 behavioral tracking software (Noldus Information Technology, Netherlands). The behavioral parameters tested in this study were distance traveled and duration of immobility at a certain coordinate.

Naïve mice were randomly distributed into three groups (*n* = 8–18 per group), which received either placebo (saline), M100907 (0.03 mg/kg), or naloxone (5 mg/kg) at designated time points. Behaviors were monitored for 60 min to evaluate drug-induced behavioral changes. The Noldus PhenoTyper system could accurately calculate the horizontal distance traveled, but not fine behaviors. For example, the system could not distinguish between the head and tail when detecting the direction of movement. Behaviors were monitored with video monitoring (e.g., distance traveled) and manual scores (e.g., jumping).

### Induction of Heroin Withdrawal

**FIGURE 1:**
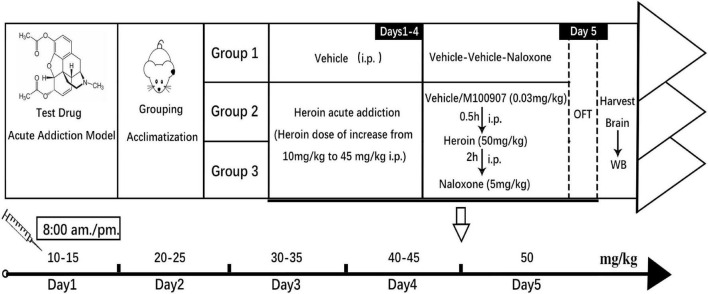
Experimental outline of the animal study. Induction of heroin dependency by administering increasing doses of heroin is shown in the graph. The MDL100907 treatment schedule is also shown.

Heroin was injected subcutaneously twice daily at gradually increasing doses (5 mg/kg each time) from 10 to 50 mg/kg by day 5. On day 5, mice were injected with saline or MDL100907 at 8:00 a.m. Thirty minutes later, mice were injected with 50 mg/kg of heroin or placebo. After 2 h, naloxone was administered to control withdrawal symptoms, including jumping (el-Kadi and Sharif, 1995). The naloxone-precipitated heroin-withdrawal mouse model is illustrated in Figure 1. Behavioral parameters such as total distance traveled and spontaneous immobility were monitored by video recording. Heroin-withdrawal behaviors after naloxone administration were monitored for 30 min. Withdrawal behaviors included wet dog shakes, body grooming, penile grooming, head shakes, paw licking, jumping (completely in the air), extended posture, rearing, and burrowing (escape digging).

### Western Blot

Mice were euthanized immediately after day 5 of the experiment, and PFC tissue was collected on ice and stored at −80°C. PFC tissues were homogenized and lysed in ice-cold 1× RIPA buffer for protein extraction, followed by centrifugation (4°C, 10,000 × *g*, 10 min). The supernatant containing total protein was transferred to a new Eppendorf tube, and the protein concentration was determined using a BCA protein detection kit. The protein samples were resolved on a 10% SDS-polyacrylamide gel by electrophoresis and transferred to a polyvinylidene fluoride membrane. The membranes were blocked with non-fat-skimmed milk (5% w/v) and washed with Tris-buffered saline with 0.1% v/v Tween-20, then probed with primary antibody (1:100) overnight at 4°C, followed by the HRP-conjugated secondary antibody at 1:10,000 dilution for 1.5 h. Protein bands were displayed with enhanced chemiluminescence substrates (Thermo Fisher Scientific Inc.) and captured using a ChemiDoc™ XRS+ imaging system (BioRad Co., United States). The density of the protein bands was quantified (gray mean value) using ImageJ software (NIH). Density measurements for 5-HT2AR were normalized to the internal control β-actin (Zhang et al., 2016). There were three independent trials for each group.

### Statistical Analysis

Statistical differences in total distance traveled and duration of immobility were analyzed using two-way repeated-measures ANOVA. All data are expressed as mean ± SEM. One-way ANOVA and Student’s *t*-test were used for statistical analysis. If statistical significance was found (*p* < 0.05), a *post hoc* Dunnett’s test or Bonferroni multiple comparison was conducted.

## Results

### MDL100907 Overdosing Impairs Locomotor Activity in Mice

**FIGURE 2:**
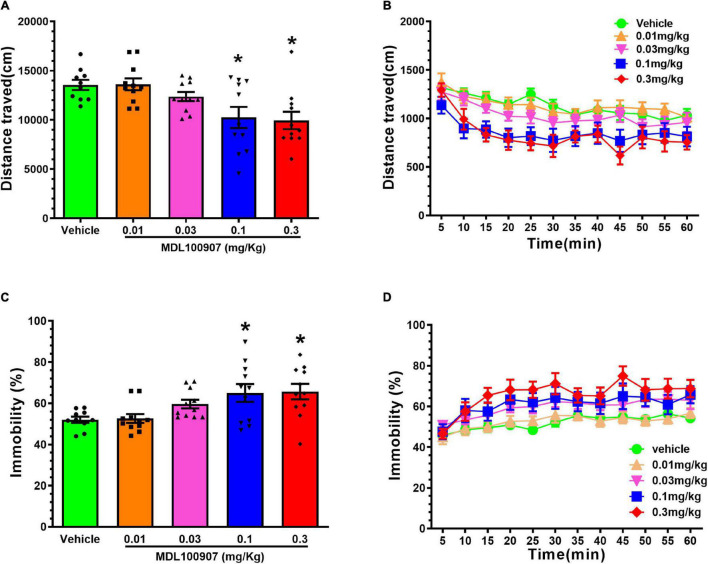
Effects of MDL100907 on locomotor activity. **(A)** Five groups of naïve mice received one of five MDL100907 doses each (i.e., 0, 0.01, 0.03, 0.1, and 0.3 mg/kg). Distance covered in 60 min. **(B)** Distance covered in a 5-min bin period. **(C)** Percentage of immobility. **(D)** Immobility in a 5-min bin period. Data are expressed as mean ± SEM; *n* = 8–18 for each group *p < 0.05.

To observe the effects of MDL100907 on locomotor activity, we first compared the effects of MDL100907 on the distance traveled and immobility duration. MDL100907 treatments at 0.1 and 0.3 mg/kg doses, but not at 0.01 and 0.03 mg/kg doses, significantly reduced locomotor activity compared with controls [*F*(4,49) = 5.457, *p* = 0.0010; Figure 2A]. MDL100907 treatment had a significant effect not only on the distance traveled [*F*(4,36) = 6.238, *p* < 0.001] and also on the 5-min time bin [*F*(11,99) = 18.895, *p* < 0.0001]; however, there was no significant effect on the treatment × time bin interaction [*F*(44,396) = 1.344, *p* = 0.077; Figure 2B].

Next, we measured the immobility duration for 1 h [*F*(4,49) = 4.667, *p* = 0.0029, one-way ANOVA *post hoc* Dunnett’s multiple comparisons; Figure 2C]. A two-way repeated-measures ANOVA on immobility, measured in every 5 min, showed no statistically significant effect on immobility duration [*F*(4,36) = 0.356, *p* = 0.838; Figure 2D]; however, the duration of MDL100907 treatment had significant effects not only on immobility duration [*F*(44,396) = 3.480, *p* < 0.0001] but also on the time × treatment interaction [*F*(4,36) = 0.356, *p* < 0.0001]. Injection of 0.3 mg/kg MDL100907 did not affect locomotor activity, so 0.03 mg/kg MDL100907 was administered subcutaneously to heroin-treated mice. We also tested whether MDL100907 affected activity/immobility in the absence of naloxone and found no difference between MDL100907 alone and control (data not shown).

### MDL100907 Relieves Naloxone-Induced Precipitated Withdrawal Symptoms in Heroin-Dependent Mice

**FIGURE 3:**
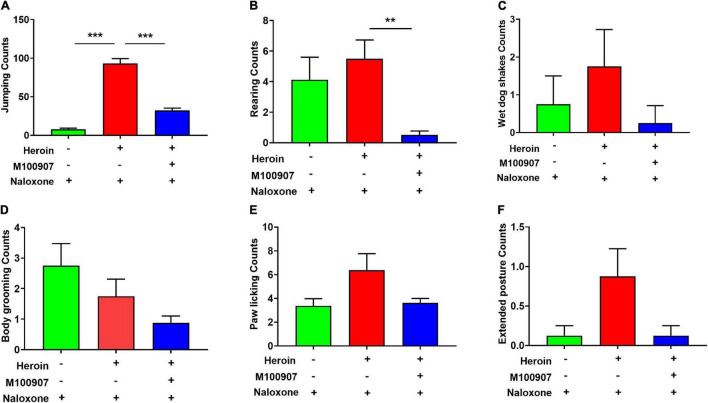
Effects of MDL100907 on naloxone-induced withdrawal symptoms. **(A)** Jumping. **(B)** Rearing counting. **(C)** Wet dog shakes. **(D)** Body grooming. **(E)** Paw licking. **(F)** Extended posture. All behaviors were monitored for 30 min. Data are expressed as mean ± SEM; *n* = 8–18 for each group **p < 0.01, ***p < 0.001.

Figure 3 shows the frequency of jumping (Figure 3A), rearing (Figure 3B), wet dog shakes (Figure 3C), body grooming (Figure 3D), paw licking (Figure 3E), and extended posture (Figure 3F) was counted for 30 min. Mice showed physical hyperactivities after heroin withdrawal, such as heavily increased rearing and jumping, compared with mice treated with saline and naloxone alone. After heroin withdrawal, MDL100907 significantly suppressed rearing and jumping (*p* < 0.05) but had no effect on the frequency of wet dog shakes, body grooming, and stretched out posture (Figure 3).

### MDL100907 Negatively Modulates Distance Moved and Mobility in Naloxone-Precipitated Withdrawal Symptoms

**FIGURE 4:**
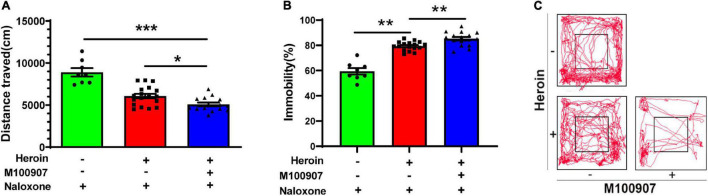
MDL100907 suppresses opioid-withdrawal symptoms. Mice were treated with increasing doses of heroin (10–50 mg/kg) for 4.5 days to develop heroin dependency. **(A,B)** MDL100907 significantly reduced the distance traveled and significantly increased immobility. Plot tracking of the open-field test is shown. **(C)** MDL100907 reduced hyperactive walking activity in heroin-exposed mice treated with naloxone, but not in heroin-exposed mice treated with saline. Data are expressed as mean ± SEM; *n*(C) = 8–18; *p < 0.05; **p < 0.01; ***p < 0.001 vs. saline.

Heroin dependency was successfully induced after 4.5 days of gradually increasing doses. MDL100907 (0.03 mg/kg) significantly restricted the distance traveled per hour compared with saline treatment [*F*(2,37) = 30.646, *p* < 0.0001; Figure 4A; movement track, Figure 4C]. MDL100907 treatment also significantly increased the duration of immobility [*F*(2,37) = 65.70, *p* < 0.01; Figure 4B] after 30 min, but not after 1 h in naloxone-precipitated heroin-withdrawn mice compared with saline treatment. MDL100907 treatment also reduced the distance moved and increased the duration of immobility (*p* < 0.05) compared with saline treatment.

### Heroin Treatment Upregulates 5-Hydroxytryptamine (Serotonin) 2A Receptor Expression and Downregulates ERK Phosphorylation in the Medial Prefrontal Cortex

**FIGURE 5:**
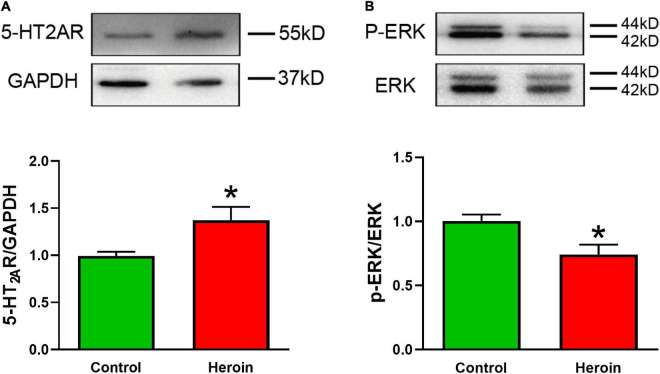
Acute heroin exposure is associated with increased 5-HT2AR expression and decreased p-ERK expression in the PFC. Mice were injected with increasing concentrations of heroin for 5 days to develop heroin dependency, and PFC tissue was collected 2.5 h after the last shot of heroin. **(A,B)** Acute heroin treatment increased 5-HT2AR protein levels and decreased the p-ERK/ERK ratio in PFC tissue. Data are expressed as mean ± SEM; *n* = 4 per group; *p < 0.05 vs. saline group.

Brain tissue was collected from acute heroin-dependent mice 2.5 h after the last heroin administration, and the levels of 5-HT2AR, ERK, and phosphorylation of extracellular signal-regulated kinase (p-ERK) in the medial prefrontal cortex (mPFC) were quantified by Western blotting. Heroin exposure correlated positively with 5-HT2AR expression [*t*(6) = 2.570, *p* < 0.05]. The p-ERK/ERK ratio in mPFC tissue [*t*(6) = 2.88, *p* < 0.05] was lower in heroin-treated mice than in saline-treated mice (Figures 5A,B).

## Discussion

People who have experienced heroin dependence are vulnerable to relapses even years into abstinence (Stewart et al., 1984; Lucey et al., 2019). Impulsive reactions to stimuli without regard for the negative consequences can contribute to relapse (Volkow et al., 2019) and are regulated by 5-HT neurotransmission (Anastasio et al., 2015; Fink et al., 2015). This study explored the influence of 5-HT2ARs on withdrawal symptoms in heroin-treated mice. We report that the 5-HT2AR antagonist MDL100907 attenuates naloxone-precipitated withdrawal symptoms. These findings suggest that 5-HT2ARs regulate behaviors related to heroin dependence and that heroin dependence might be treated by pharmacological activation of 5-HT2ARs.

We found that the 5-HT2AR antagonist MDL100907 alleviates naloxone-induced precipitated withdrawal symptoms in heroin-exposed mice. Lower doses of MDL100907 did not affect baseline motor activity, whereas higher doses inhibited heroin-withdrawal symptoms. In line with these findings, another study showed that higher doses of MDL100907 reduce nicotine-withdrawal symptoms in rats (Malin et al., 2019). Previous studies have demonstrated that naloxone-precipitated heroin withdrawal is characterized by jumping, whereas others have suggested that these behaviors are not specific to heroin withdrawal and may be caused by a floor effect (Pang et al., 2016).

The heroin-withdrawal symptoms might be caused by increased long-lasting responses of neurons innervating the nucleus accumbens, dopaminergic neurons in the ventral tegmental area (VTA), and glutamatergic neurons in the PFC and basolateral amygdala (Russo and Nestler, 2013). This suggests that heroin derivatives activate MOR, thereby inducing locomotor hyperactivity and increasing dopaminergic neurotransmission. The VTA-nucleus accumbens circuit is critical for reward recognition and includes the mPFC, amygdala, hippocampus, and other regions regulated by dopaminergic neurons. Previous studies have revealed that dopaminergic neurons in the nucleus accumbens can secret glutamate and γ-aminobutyric acid (GABA), which may contribute to dependence on opioids (Hnasko et al., 2012; Tritsch et al., 2012). 5-HT2ARs are widely distributed in intermediate inhibitory neurons, major VTA neurons, dorsomedial PFC neurons, and BLA neurons (Kreek et al., 2012). The 5-HT2AR is expressed in GABA-ergic neurons in the midbrain, substantia nigra, and VTA, but not in dopaminergic neurons (Kuehn, 2013). Furthermore, the 5-HT2AR antagonist MDL100907 reduces the firing rate of dopaminergic neurons in the mesolimbic system (Lucey et al., 2019). These results suggest that MDL100907-mediated blocking of 5-HT2ARs may inhibit dopamine neurotransmission, which may inhibit heroin-withdrawal symptoms in mice. Neuronal excitation is crucial to heroin withdrawal and may be blocked by MDL100907 treatment. Further experiments are warranted to delineate the underlying mechanisms.

Immunoblot analysis revealed that 5-HT2AR expression was upregulated in PFC tissue from heroin-dependent mice. The PFC is a key brain region associated with opioid addictive behavior, so the increased 5-HT2AR expression we observed suggests a crucial link to neural adaptation upon heroin exposure. However, the functional significance of 5-HT2AR overexpression in heroin addiction remains elusive. Elucidating the subcellular distribution and receptor signal transduction of 5-HT2AR will help understand the regulatory mechanisms of heroin dependence. Interestingly, we observed reduced ERK phosphorylation in PFC tissue from heroin-dependent mice, although 5-HT2AR activation upregulates p-ERK1/2 expression. Heroin triggers multiple cellular signaling cascades and, together with its metabolites, can have diverse effects in different brain regions, neural networks, and cell types. This variability might explain the inconsistency between the heroin-mediated increase in 5-HT2AR expression and reduced p-ERK accumulation we observed. It was recently reported that morphine also downregulates p-ERK/ERK. This effect was induced by the D1R–ERK–CREB pathway in the mPFC, and the D1R antagonist SCH-23390 reversed morphine-induced attention dysfunction and morphine-withdrawal symptoms in mice (Yin et al., 2021).

The 5-HT2AR-mediated heroin withdrawal may involve dopamine neurotransmission. 5-HT2ARs control dopamine outflow to augment dopamine synthesis and excitation (Schmidt et al., 1992; Lucas and Spampinato, 2000; Porras et al., 2002; Alex and Pehek, 2007). However, selective blockade of 5-HT2ARs by SR46349B did not affect the cocaine-induced increase in dopamine outflow in the nucleus accumbens and striatum (Auclair et al., 2004). Furthermore, MDL100907 did not affect basal and cocaine-augmented dopamine outflow into the mPFC (Bonaccorso et al., 2002; Fletcher et al., 2002; Li et al., 2005; Berg et al., 2008), whereas coadministration of MDL100907 and the 5-HT2CR antagonist lorcaserin suppressed cocaine-seeking behavior (Anastasio et al., 2020). 5-HT receptors control ascending dopamine pathway activity and the neurochemical and behavioral responses to cocaine (Alex and Pehek, 2007; Berg et al., 2008; Filip et al., 2012; Devroye et al., 2013; Howell and Cunningham, 2015; Sholler et al., 2019). However, their impact on cocaine-mediated increases in dopamine production in the mPFC remains unclear. Dopaminergic signaling also contributes to opioid addiction by modulating the hypothalamic–pituitary–adrenal (HPA) axis. The relationship between cocaine use, the HPA axis, and opioid use is well-established (Nava et al., 2006; Picetti et al., 2013; Walter et al., 2013), and it will be interesting to elucidate the role of the HPA axis on heroin dependence in a future study.

Taken together, our data have shown that naloxone-induced heroin-withdrawal symptoms can be attenuated by MDL100907. Acute heroin addiction increased 5-HT2AR expression in the PFC, suggesting that 5-HT2AR might be an efficient therapeutic target for treating heroin use disorders.

## Data Availability Statement

The raw data supporting the conclusions of this article will be made available by the authors, without undue reservation.

## Ethics Statement

The animal study was reviewed and approved by the Institutional Animal Care and Use Committee (IACUC).

## Author Contributions

GP and XT conceived and designed the study. BL, JJ, LZ, QS, JL, and YL conducted the experiments, collected the data, and prepared the manuscript. All authors contributed to the article and approved the submitted version.

## Conflict of Interest

The authors declare that the research was conducted in the absence of any commercial or financial relationships that could be construed as a potential conflict of interest.

## Publisher’s Note

All claims expressed in this article are solely those of the authors and do not necessarily represent those of their affiliated organizations, or those of the publisher, the editors and the reviewers. Any product that may be evaluated in this article, or claim that may be made by its manufacturer, is not guaranteed or endorsed by the publisher.
